# Inhibition of CCR8 attenuates Ang Ⅱ-induced vascular smooth muscle cell injury by suppressing the MAPK/NF-κB pathway 

**DOI:** 10.22038/ijbms.2022.64524.14191

**Published:** 2022-09

**Authors:** Lisi Liao, Di Song, Bobo Shi, Ming Chen, Linghu Wu, Jinfeng Xu, Fajin Dong

**Affiliations:** 1 The Second Clinical Medical College, Jinan University; Department of Ultrasound, The First Affiliated Hospital, Southern University of Science and Technology; Department of Ultrasound, Shenzhen People’s Hospital, Shenzhen 518020, Guangdong, China; # These authors contributed eqully to this work

**Keywords:** CCR8, Hypertension, Inflammation, Insulin resistance, MAPK/NF-κB signaling, Oxidative

## Abstract

**Objective(s):**

Hyperinsulinemia, secondary to insulin resistance, may lead to vascular smooth muscle cell dysfunction. In the present research, we aimed to investigate the effect of Chemokine receptor 8 (CCR8) on angiotensin II (Ang II)-induced dysfunction of vascular smooth muscle cells (VSMCs) and to explore the underlying molecular mechanism.

**Materials and Methods:**

The expression of CCR8 was analyzed in diabetics and normal people by RT-PCR and ELISA. CCK-8 assay and transwell were used to explore cell proliferation and migration, and ELISA was used to measure the content of IL-6 and TNF-α. Reactive oxygen species (ROS) kit was employed to measure ROS generation.

**Results:**

The results revealed that CCR8 was highly expressed in diabetics and Ang Ⅱ-induced VSMCs. Further studies found that interfering with the expression of CCR8 significantly reduced the production of ROS and the levels of inflammatory factors in AngⅡ-induced VSMCs. Interfering with CCR8 increased the glucose uptake induced by AngⅡ+IR. More importantly, inhibition of CCR8 alleviated Ang II-induced dysfunction of VSMCs. Inhibition of CCR8 inactivated the MAPK/NF-κB signaling pathway.

**Conclusion:**

Inhibition of CCR8 attenuates Ang II-induced VSMCs injury by inhibiting the MAPK/NF-κB pathway. CCR8 may be a new biomarker related to hypertension and insulin resistance and is a new target for the treatment of human cardiovascular diseases.

## Introduction

Hypertension is one of the most common chronic diseases and the main risk factor for cardiovascular and cerebrovascular diseases (1). More and more studies confirmed that people with essential hypertension have significant insulin resistance (2). The appropriate concentration of insulin facilitates vasodilation and maintains blood circulation. However, hypertension and insulin resistance often co-exist and interact with each other, playing a joint role in multiple chronic diseases. Insulin resistance is a condition in which the efficiency of insulin to promote glucose uptake and utilization decreases for various reasons, and the body secretes excessive insulin to produce hyperinsulinemia, which maintains blood glucose stability. Insulin resistance or reduced insulin sensitivity is an important feature of metabolic syndrome, which is associated with obesity, impaired glucose tolerance, inflammation, and hypertension (3, 4). Therefore, in recent years, the concept of prevention and treatment of hypertension has undergone great changes, lowering blood pressure while improving insulin resistance has become a new approach to treating hypertension (5).

Chemokine receptor 8 (CCR8) is a G protein-coupled receptor and is usually expressed in immune cells, including lymphocytes, macrophages, and monocytes (6, 7). In mammals, CCR8 is usually selectively activated by CC chemokine 1 (CCL1) (8). Islam *et al*. reported that CCR8 is a functional receptor of CCL18 (9). CCL1/CCR8 is an inducer in immune cells, which promotes the activation and recruitment of lymphocytes and macrophages (10). CCL1/CCR8 play an important role in inflammatory diseases, such as dermatitis, asthma, peritonitis, and hepatitis. In addition, CCR8 is involved in immune homeostasis (11). In cardiovascular diseases, CCR8 is expressed in endothelial cells and macrophages, which stimulates the migration of endothelial cells (12). However, the role and mechanism of CCR8 in hypertension and insulin resistance are still unclear and need to be further explored.

In the present research, we investigated the effects and molecular mechanism of CCR8 on the angiotensin II (Ang II)-induced vascular smooth muscle cells (VSMCs) proliferation and migration, as well as oxidative stress, inflammation, and apoptosis *in vitro* and to provide a valid theoretical basis for clinical treatment of hypertension. 

## Materials and Methods

***Collection of blood samples***

From October 2018 to December 2019, 32 cases of diabetics were treated, including 18 males and 14 females, aged 42–70 years (average 53 years). The patients were treated in our hospital. In addition, fresh blood samples were collected from all these patients, and blood samples from 32 volunteers without a history of cardiovascular disease were taken as controls. The study was approved by the local ethics committee and received written consent from all selected patients and volunteers.

***Extraction of total RNA from blood samples***

In this experiment, a blood total RNA extraction kit (catalog number: DP433, Tiangen Biotechnology, Beijing, China) was used to extract RNA from fresh blood. The specific operation is as follows: add 1 ml of erythrocyte lysis solution to 200 μl of whole blood, and incubate for 15 min until the erythrocytes are lysed. After centrifugation, the supernatant was removed to obtain a leukocyte pellet. Lysate RL was added to the leukocyte pellet to completely lyse the cells. The solution was transferred to filter cartridge CS for filtration. An equal volume of 70% ethanol was added to the collected filtrate and transferred to the adsorption column CR2 again. Add protein-free solution RW1 to the centrifuged adsorption column CR2. Centrifuge the adsorption column again and discard the waste liquid. Add the DNase working solution to the center of the adsorption column CR2, and then add the protein-removing solution RW1 after incubation at room temperature. Add rinse solution to adsorption column CR2, centrifuge, and air dry. Use DNase-Free water to dissolve the RNA on the adsorption column to obtain the total RNA in the sample.

***Determination of CCR8 content***

In this experiment, human chemokine CC receptor 8 (CCR8) ELISA kit (catalog number: BD-EL-H0704, Bidi Bio, Nanjing, China) was used to detect the level of CCR8 in the serum of clinical samples. Briefly, add the sample diluent and the test sample to the ELISA-coated plate, and dilute the test sample by 5 times. The samples were incubated at 37 °C for 30 min and washed with washing solution. Enzyme labeling reagents were added to each well, incubated, and washed. Add color developer A and color developer B to each well in sequence, and after mixing, the color develops in the dark for 15 min. The reaction was terminated by adding a stop solution, and the absorbance (OD value) of each well was measured at 450 nm.

***Cell culture***

Vascular smooth muscle cells (VSMC) were purchased from Cell Applications (San Diego, CA, USA). Cells were cultured in DMEM medium (ThermoFisher, Massachusetts, USA) containing 10% FBS (ThermoFisher, Massachusetts, USA) and grown at 37 °C in a humidified incubator with 5% CO_2_.

***Cell transfection and treatment***

siRNA and si-CCR8 were designed and synthesized by Tsingke Biotech Co., Ltd. (Beijing, China). The plasmid vectors were transfected into VSMCs using Lipofectamine 2000 (Invitrogen, USA) according to the manufacturer’s instructions. After 24 hr of cell transfection, the transfection efficiency of CCR8 in cells was determined.

For Ang II treatment, cells were stimulated with 100 nM Ang II (Sigma-Aldrich, USA) for 6 hr, 12 hr, 24 hr, and 48 hr, respectively. For Ang II treatment of transfected cells, cells receiving or not receiving transfection were stimulated with 100 nM Ang II for 48 hr. Cells transfected and stimulated with Ang II were collected for subsequent experiments.

For P79350 treatment, cells receiving or not receiving transfection were treated with 50 μM of p38 MAPK agonist P79350 (Invitrogen, Thermo Fisher Scientific, USA) for 1 hr. Cells were collected and used for subsequent experiments.

***CCK-8 assay***

The VSMCs were inoculated into a 24-well plate and cultured at 37 °C for 24 hr. 2-(2-methoxy-4-nitrophenyl)-3-(4-nitrophenyl)-5-(2,4-disulfobenzene (WST-8) (10 μl) was added to the plate well for 24 hr. The OD value at 450 nm was determined.

***Transwell assay***

Cell migration and invasion were evaluated using Transwell migration chambers (8-µm pore size; Millipore, Boston, MA, USA) pre-coated with or without a layer of Matrigel (excreta cellular matrix gel; Sigma, St. Louis, MO, USA). Cells were seeded into the upper compartment of the invasion chamber filled with FBS-free culture medium, the lower chamber contained a 700 µl RPMI1640 medium with 10% FBS, which served as a chemoattractant. After incubation at 37 °C for 48 hr, the cells that remained in the upper compartment were removed by cotton swabs. The migrated and invaded cells were washed with PBS, fixed with 4% paraformaldehyde, stained with 0.1% crystal violet, and counted by light microscopy.

***Glucose consumption assay***

In this experiment, a glucose kit (Nanjing Jiancheng, Nanjing, China) was used to detect glucose consumption in cells. Briefly, cells grown to 80% were treated with various concentrations of insulin (1-5 µmol/l) for 30 min. Cells were washed with PBS to remove insulin, and the medium was replaced with DMEM containing 11.1 mmol/l glucose and 0.2% BSA. After culturing for 24 hr, 10 μl of the medium was taken to measure the glucose consumption of the cells.

***Glycogen content assay***

In this experiment, a glycogen assay kit (Solarbio, Beijing, China) was used to detect the content of glycogen in cells. Briefly, test cells were treated with 5 µmol/l insulin for 20 min. The collected cells were centrifuged to obtain the cell pellet. The extraction solution was added to cells and cells were sonicated. The cells were continuously boiled for 20 min, centrifuged and the supernatant was collected for testing. Glycogen content in cells was determined using the sulfate-anthrone colorimetric method according to the manufacturer’s instructions.

***RTqPCR***

Total RNA was extracted according to RNA extraction kit instructions. Then the reverse transcription was performed according to the instructions of the reverse transcription kit, and the transcription levels of genes were detected by the RT-PCR kit with the template of the cDNA. The reaction conditions were as follows: 95 °C 10 min, 95 °C 20 sec, 60 °C 1 min, and 32 cycles were amplified.

***Western blotting***

Cells in each group were collected and lysed with cell lysate. The supernatant was collected by centrifugation at 4 °C for 20 min with 10000×g. The concentration of extracted protein was according to the BCA kit, and protein was separated by SDS-PAGE. The protein was electrotransferred to the PVDF membrane. The PVDF membranes were dyed with Lichunhong dye solution. The membranes were cleaned three times with TBST solution for 10 min each time. Skimmed milk was sealed, and 3 ml skimmed milk was added to the antibody incubator. The membranes were sealed for 1 hr at room temperature. TSBT solution cleaning membrane was done 3 times, 10 min each time; first antibody incubating was overnight at 4 °C environment, TBST solution cleaning membrane was done 3 times, 10 min each time; the second antibody was incubated at room temperature for 1 hr. Finally, an appropriate amount of luminescent solution (A and B liquid volume was mixed) was used to incubate the membrane for 3 min, the gel imaging system was exposed, and Quantity-One software was used to analyze the protein content. Antibodies including anti-CCR8, anti-COX-2, anti-iNOS, anti-ERK1/2, anti-p-ERK1/2, anti-JNK, anti-p38 MAPK, anti-p-p38 MAPK, anti-NFκB p65, and anti-p-NFκB p65 were all obtained from Invitrogen Biotechnology (Massachusetts, USA). anti-GAPDH was purchased from Abcam (Cambridge, UK).

***Statistical analyses***

All statistical analyses were performed using SPSS 22.0, (Chicago, IL, USA). The data are presented as the mean ± SD. Student’s t-test was used to compare the two groups. When* P*<0.05, the difference was considered to be statistically significant.

## Results

***Expression of CCR8 in diabetic patients was higher than in normal people and increased in high Ang*** ***Ⅱ*** ***-induced VSMCs***

**Figure 1:**
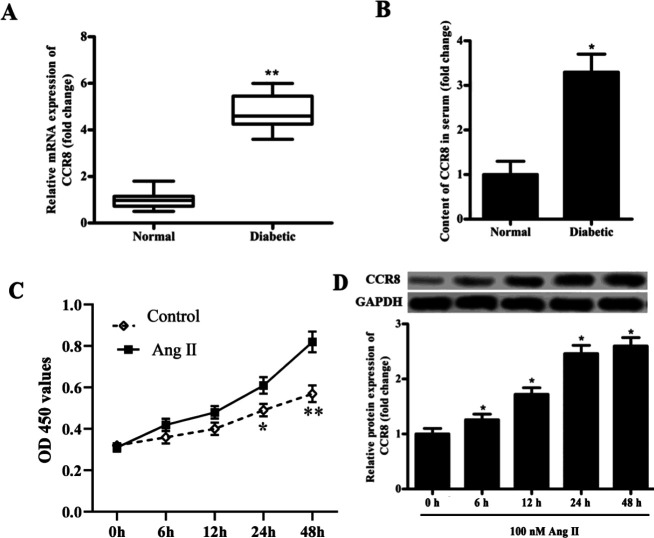
The expression of CCR8 in diabetic patients was higher than that in normal people, and increased in Ang Ⅱ-induced VSMCs. (A) RT-qPCR was used to measure the mRNA expression of CCR8 in diabetic patients and normal people. (B) ELISA was used to measure the relative content of CCR8 in serum of diabetic patients and normal people. (C) The CCK-8 was used to detect cell proliferation of VSMCs. (D) The protein expression of CCR8 in VSMCs treated with 100 nM Ang Ⅱ at 0 hr, 6 hr, 12 hr, 24 hr and 48 hr. “*” means compared with the normal or control group at *P*<0.05, “**” means compared with the normal or control group at *P*<0.01. GAPDH was used as an invariant internal control for calculating protein fold-changes

CCR8: Chemokine receptor 8; Ang Ⅱ: Angiotensin II; VSMCs: Vascular smooth muscle cells

The clinical characteristics of normal and diabetes participants are shown in Table 1. The mRNA expression of CCR8 in 32 diabetic patients and 32 normal people was analyzed. The results showed that the expression of CCR8 in the serum of diabetic patients was significantly higher than that in normal people (Figure 1A). Besides, the results of ELISA showed that the content of CCR8 in the serum of diabetic patients was significantly increased compared with that in normal people (Figure 1B). The proliferation of VSMCs was elevated by Ang Ⅱ in a time-dependent manner (Figure 1C). What’s more, the protein expression of CCR8 increased significantly in high Ang Ⅱ-induced VSMCs and showed a significant time-dependence (Figure 1D).

***si-CCR8 alleviates Ang*** ***Ⅱ-*** ***induced dysfunction in VSMCs***

**Figure 2:**
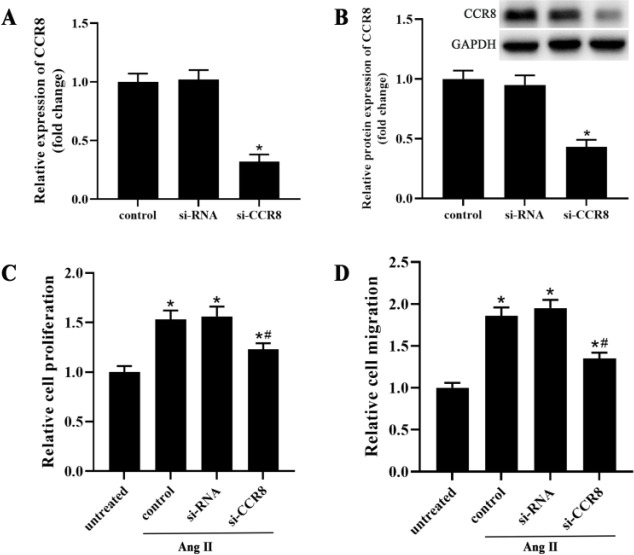
si-CCR8 alleviates high Ang Ⅱ-induced dysfunction in VSMCs. The VSMCs were transfected with the control si-RNA (si-RNA group) or si-CCR8 (si-CCR8 group) for 24 hr. (A and B) qPCR and Western blot were performed to confirm the transfection efficiency. (C) CCK-8 was used to evaluated the cell proliferation of VSMCs. (D) The cell migration was measured by transwell assay. “*” means compared with the untreated group at *P*<0.05, and “#” means compared with the control group at *P*<0.05. GAPDH was used as an invariant internal control for calculating protein fold-changes

CCR8: Chemokine receptor 8; Ang Ⅱ: Angiotensin II; VSMCs: Vascular smooth muscle cells

The VSMCs were transfected with the control si-RNA (si-RNA group) or si-CCR8 (si-CCR8 group) for 24 hr. As shown in Figures 2A and B, the mRNA and protein expression of CCR8 was significantly decreased in the si-CCR8 group compared with the control and si-RNA groups (Figure 2A and B). To investigate the effect of CCR8 on VSMCs dysfunction, the cell proliferation and migration of VSMCs were examined. The results showed that the proliferation and migration of VSMCs were markedly elevated after Ang Ⅱ treatment (Figure 2C and D). However, the silence of CCR8 reversed this effect of increased proliferation and migration of VSMCs induced by Ang Ⅱ. Inhibition of CCR8 remarkably suppressed the high Ang Ⅱ-induced VSMCs proliferation and migration, compared with the control group (Figure C and D).

***si-CCR8 alleviates high Ang*** ***Ⅱ-*** ***induced ROS generation and inflammation in VSMCs***

**Figure 3:**
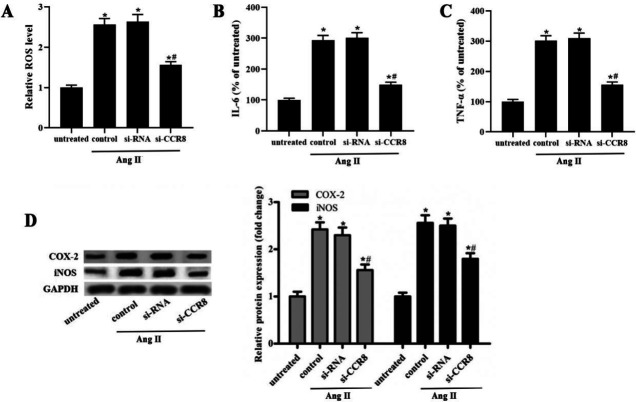
si-CCR8 alleviates alleviates high Ang Ⅱ-induced ROS generation and inflammation in VSMCs. The VSMCs were transfected with the control si-RNA (si-RNA group) or si-CCR8 (si-CCR8 group) for 24 hr. (A) The ROS generation in the untreated, control, si-RNA and si-LRRK2 group. (B) The IL-6 level in the untreated, control, si-RNA and si-CCR8 group. (C) The TNF-α level in the untreated, control, si-RNA and si-LRRK2 group. (D) The protein expression of COX-2 and iNOS in the untreated, control, si-RNA and si-CCR8 group. “*” means compared with the untreated group at *P*<0.05, and “#” means compared with the control group at *P*<0.05. GAPDH was used as an invariant internal control for calculating protein fold-changes

CCR8: Chemokine receptor 8; Ang Ⅱ: Angiotensin II; ROS: Reactive oxygen species; VSMCs: Vascular smooth muscle cells; TNF-α: Tumor necrosis factor; COX-2: Cyclooxygenase-2; iNOS: Inducible nitric oxide synthase; GAPDH: Glyceraldehyde-3-phosphate dehydrogenase

**Figure 4:**
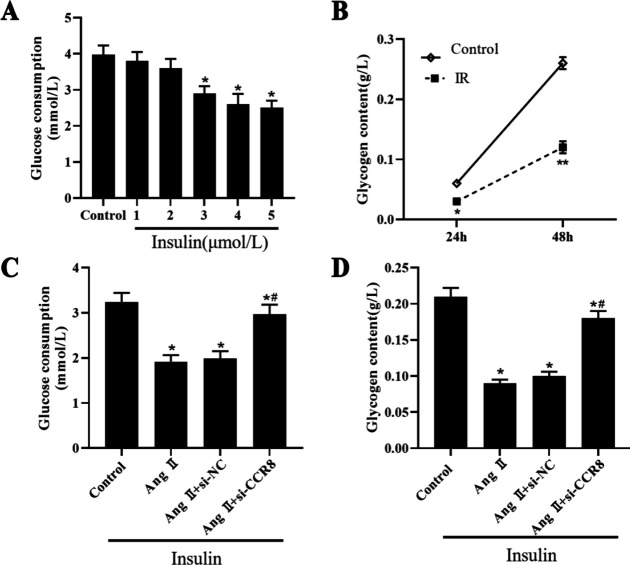
Inhibition of CCR8 increased the Ang Ⅱ+IR-induced glucose uptake. (A) The glucose consumption of insulin on VSMCs. (B) The glycogen content of IR treatment on VSMCs. (C) The glucose consumption in the control, Ang II, Ang II+si-RNA and Ang II+si-CCR8 group. (D) The glycogen content in the control, Ang II, Ang II+si-RNA and Ang II+si-CCR8 group. “*” means compared with the control group at *P*<0.05, and “#” means compared with the control group at *P*<0.05. GAPDH was used as an invariant internal control for calculating protein fold-changes

CCR8: Chemokine receptor 8; Ang Ⅱ: Angiotensin II; VSMCs: Vascular smooth muscle cells

We next explored the effect of CCR8 on ROS generation and inflammation in Ang Ⅱ-induced VSMCs. The results showed that the ROS content in the insulin treatment group was markedly enhanced, compared with the untreated group (Figure 3A). Inhibition of CCR8 significantly inhibited ROS generation in Ang Ⅱ-induced VSMCs, compared with control (Figure 3A). As we all know, inflammation is one of the key factors to promote the progress of cardiovascular disease (13). The results showed that silence of CCR8 significantly reduced the level of IL-6 and TNF-α in Ang Ⅱ-induced VSMCs (Figure 4B and C). Furthermore, the expression of COX-2 and iNOS was markedly increased in Ang Ⅱ-induced VSMCs, while si-CCR8 significantly decreased the COX-2 and iNOS expression (Figure 4D).

***Inhibition of CCR8 increased the Ang*** ***Ⅱ*** ***+IR-induced glucose uptake***

As shown in Figure 4, glucose consumption was decreased with the increase in insulin concentration (Figure 4A). Then, the glycogen content of VSMCs after IR induction for 24 hr and 48 hr was evaluated using the anthrone-sulfuric acid method. The results showed that the glycogen content in IR-induced VSMCs was markedly decreased compared with the control group (Figure 4B). Ang II affects insulin signaling in various cells, and Ang II inhibits insulin-stimulated glucose uptake. Here, we investigated the role of CCR8 in Ang II-induced insulin resistance in VSMCs. The data revealed that Ang II treatment reduced the insulin-induced glucose consumption and glycogen content of VSMCs (Figure 4C and D). However, inhibition of CCR-8 restored the decrease of glucose consumption and glycogen content caused by Ang II (Figure 4C and D).

***Inhibition of CCR8 inactivates the MAPK/NFκB signaling pathway in Ang II-induced VSMCs***

**Figure 5:**
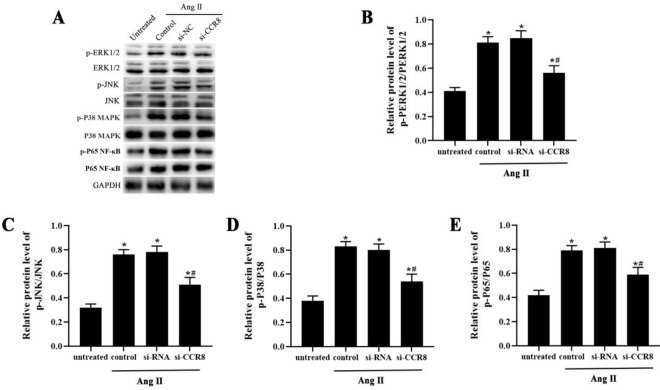
Inhibition of CCR8 inactivates the MAPK/NF κB signaling pathway in Ang II-induced VSMCs. (A-E) The protein expression of ERK1/2, p-ERK1/2, JNK, p-JNK, p38 MAPK, p-P38, nuclear NF-κB/p65 and p-P65 in control, si-RNA and si-LRRK2 group were measured by western blot. “*” means compared with the untreated group at* P*<0.05, and “#” means compared with the control group at *P*<0.05. GAPDH was used as an invariant internal control for calculating protein fold-changes

CCR8: Chemokine receptor 8; Ang Ⅱ: Angiotensin II; VSMCs: Vascular smooth muscle cells; MAPK: Mitogen-activated protein kinase

MAPK/NFκB signaling pathway plays a critical role in VSMCs dysfunction. To evaluate whether CCR8 inhibition affects MAPK/NF-κB activity and impacts Ang II-induced VSMCs pathology, western blotting was conducted to detect ERK1/2, JNK, p38 MAPK, and nuclear NF-κB/p65 protein expression. As expected, the protein expression of p-ERK1/2, p-JNK, p-p38 MAPK, and nuclear p-p65 was significantly decreased in the si-CCR8 group compared with the control group and si-RNA group (Figure 5A-E). 

***si-CCR8 alleviates Ang II-induced ROS generation and inflammation in VSMCs*** **through** ***MAPK/NFκB signaling pathway***

**Figure 6:**
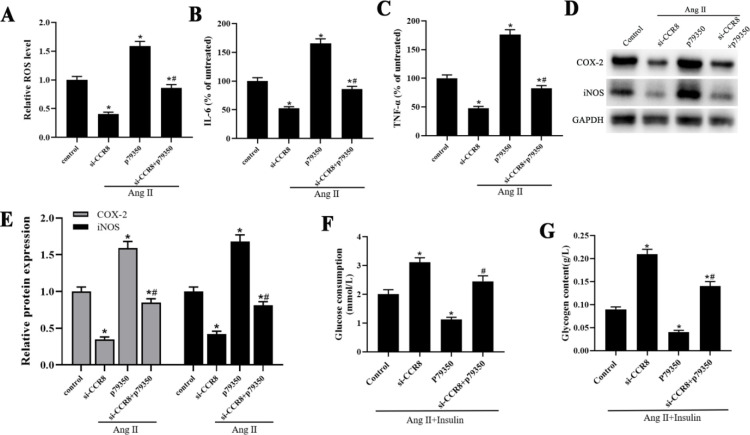
si-CCR8 alleviates Ang II-induced ROS generation and inflammation in VSMCs through MAPK/NF κB signaling pathway. (A) The ROS generation in the untreated, control, si-RNA and si-LRRK2 group. (B) The IL-6 level in the untreated, control, si-RNA and si-LRRK2 group. (C) The TNF-α level in the untreated, control, si-RNA and si-LRRK2 group. (D) The protein expression of COX-2 and iNOS in the untreated, control, si-RNA and si-LRRK2 group. “*” means compared with the untreated group at* P*<0.05, and “#” means compared with the si-CCR8 group at *P*<0.05. GAPDH was used as an invariant internal control for calculating protein fold-changes

CCR8: Chemokine receptor 8; Ang Ⅱ: Angiotensin II; VSMCs: Vascular smooth muscle cells; ROS: Reactive oxygen species; MAPK: Mitogen-activated protein kinase

The p38MAPK signaling pathway agonist P79350 was employed to investigate whether si-CCR8 affects Ang Ⅱ-induced ROS generation and inflammation in VSMCs through MAPK/NFκB signaling pathway. As shown in Figure 6, si-CCR8 significantly decreased ROS generation, release of IL-6 and TNF-α, and protein expression of COX-2 and iNOS, however, P79350 had the opposite effect (Figure 6A-E). Besides, si-CCR8+P79350 reversed the effect of si-CCR8 on ROS generation, release of IL-6 and TNF-α, and protein expression of COX-2 and iNOS in Ang Ⅱ-induced VSMCs (Figure 6A-E).

GAPDH: Glyceraldehyde-3-phosphate dehydrogenase

**Figure 7:**
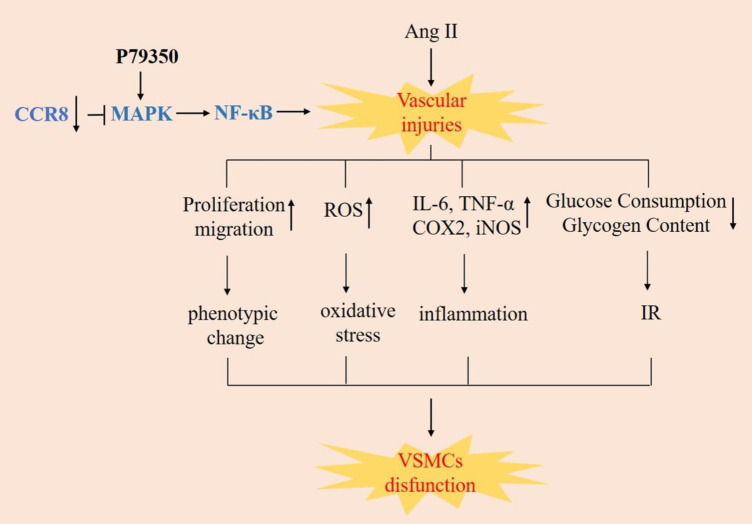
si-CCR8 alleviates Ang II-induced cell injury in VSMCs through MAPK/NF κB signaling pathway. The presetation image of graphical abstract of this study

CCR8: Chemokine receptor 8; Ang Ⅱ: Angiotensin II; VSMCs: Vascular smooth muscle cells; MAPK: Mitogen-activated protein kinase

## Discussion

Studies have reported that VSMCs have been used in various cardiovascular disease models, including hypertension, hyperlipidemia, diabetes, and insulin resistance. In the present research, we found that the expression of CCR8 was elevated in diabetic patients and Ang Ⅱ-induced VSMCs. Furthermore, inhibition of CCR8 alleviated Ang Ⅱ-induced inflammation and dysfunction by inhibiting the MAPK/NF-κB signaling pathway. It suggests that inhibition of CCR8 protects VSMCs from Ang Ⅱ-induced dysfunction and inflammation by regulating MAPK/NF-κB signaling pathway.

One of the main pathological features of hypertension is the abnormal proliferation and migration of vascular smooth muscle cells (14). In this study, Ang Ⅱ treatment increased the proliferation and migration of VSMCs. However, inhibition of CCR8 suppressed Ang Ⅱ-induced proliferation and migration in VSMCs. It is reported that inflammation plays an essential role in the occurrence and development of cardiovascular diseases, such as atherosclerosis and diabetes (15, 16). CCR8 is a cytokine expressed by activated T lymphocytes and a key factor in cell trafficking (17, 18). Therefore, CCR8 is considered to be closely related to inflammatory response and plays an important role in inflammatory diseases (19). In this study, we used Ang Ⅱ to stimulate VSMCs and found that the expression level of CCR8 was up-regulated, and the level of pro-inflammatory cytokines was increased. Similarly, Marian *et al*. found that CCR8 is highly expressed in VSMCs induced by proinflammatory agents, and the CCL1-CCR8 axis promotes atherosclerosis by inhibiting the production of IL-10 and recruitment and function of Treg (20). These results suggest that CCR8 may be involved in the occurrence and development of cardiovascular disease through the regulation of inflammatory response.

The MAPK cascade is an evolutionarily conserved signaling module consisting of protein-serine/threonine kinase family proteins, including ERK1/2, JNK, and p38 MAPK (21). NF-κB is an essential downstream transcription factor for MAPK activation in vascular smooth muscle cells (22). MAPK/NF-κB signaling pathway can regulate various cellular processes including proliferation, differentiation, inflammation, survival, and apoptosis by translating environmental information into appropriate responses through phosphorylation of substrate proteins (23). These signaling pathways are also involved in a variety of pathological processes in vascular smooth muscle cells. Previous studies have shown that inhibition of the ERK1/2 signaling pathway can inhibit Ang Ⅱ-induced VSmcs proliferation, thus suggesting a regulatory role of MAPKs on Ang Ⅱ-induced VSmc injury (24). Oxidative stress and inflammation are important factors associated with VSmc dysfunction; therefore, any factor that increases oxidative stress and inflammation may lead to VSmc injury (25). ERK, JNK, p38MAPK, and NF-κB are all ROS-sensitive regulators (26). In addition, ROS production may be mediated through activation of the MAPK/nf-κB signaling pathway (27). Inhibition of ERK1/2, JNK, or p38 MAPK has been reported to attenuate ROS accumulation-induced VSmc injury (26, 28). Thus, the activation of MAPKs and ROS production may be mutually facilitated under pathological conditions. Furthermore, there is a causal relationship between activation of the MAPK/NF-κB signaling pathway and inflammation. It has been shown that ERK1/2 and NF-κB activity can increase the expression of IL-6 and MCP-1 in vascular smooth muscle cells (26), and these findings are consistent with the results published by Wronkowitz *et al.* (29). In the present study, 100 nm insulin stimulation of VSmcs resulted in VSmc dysfunction with increased oxidative stress, inflammation, and apoptosis. Notably, mechanistic studies further showed that the expression levels of p-ERK1/2, p-JNK, p-p38MAPK, and NF-κB/p65 were significantly up-regulated. Thus, the present study suggests that activation of the MAPK/NF-κB signaling pathway may play an important role in exacerbating the harmful effects of hyperinsulinemia on VSMCs. Furthermore, inhibition of CCR8 reduced the expression levels of p-ERK1/2, p-JNK, p-p38MAPK, and p-p65. MAPK/NF-κB signaling pathway agonist P79350 reversed the effect of CCR8 on Ang Ⅱ-induced VSMC ROS production and inflammation. These data suggest that inhibition of CCR8 reduced Ang Ⅱ-induced ROS production and inflammation through the MAPK/NF-κB signaling pathway in VSMCs.

## Conclusion

In summary, knockdown of CCR8 attenuated Ang II-induced vascular smooth muscle cell injury by inhibiting the MAPK/NF-κB signaling pathway (Figure 7). It suggests that CCR8 may be a new biomarker related to hypertension and insulin resistance, and is a new target for the treatment of human cardiovascular diseases.

## Authors’ Contributions

JFX and FJD Designed the experiments; LSL, DS, BBS, MC, LHW, and JFX Carried out the experiments; FJD Performed the statistical analysis and wrote the paper.

## Funding

This project was supported by the Commission of Science and Technology of Shenzhen, China (GJHZ20200731095401004).

## Availability of Data and Material

All datasets for this study are included in the manuscript/supplementary files.

## Conflicts of Interest

The authors declare no conflicts of interest. 
